# Collocated observations of cloud condensation nuclei, particle size distributions, and chemical composition

**DOI:** 10.1038/sdata.2017.3

**Published:** 2017-03-14

**Authors:** Julia Schmale, Silvia Henning, Bas Henzing, Helmi Keskinen, Karine Sellegri, Jurgita Ovadnevaite, Aikaterini Bougiatioti, Nikos Kalivitis, Iasonas Stavroulas, Anne Jefferson, Minsu Park, Patrick Schlag, Adam Kristensson, Yoko Iwamoto, Kirsty Pringle, Carly Reddington, Pasi Aalto, Mikko Äijälä, Urs Baltensperger, Jakub Bialek, Wolfram Birmili, Nicolas Bukowiecki, Mikael Ehn, Ann Mari Fjæraa, Markus Fiebig, Göran Frank, Roman Fröhlich, Arnoud Frumau, Masaki Furuya, Emanuel Hammer, Liine Heikkinen, Erik Herrmann, Rupert Holzinger, Hiroyuki Hyono, Maria Kanakidou, Astrid Kiendler-Scharr, Kento Kinouchi, Gerard Kos, Markku Kulmala, Nikolaos Mihalopoulos, Ghislain Motos, Athanasios Nenes, Colin O’Dowd, Mikhail Paramonov, Tuukka Petäjä, David Picard, Laurent Poulain, André Stephan Henry Prévôt, Jay Slowik, Andre Sonntag, Erik Swietlicki, Birgitta Svenningsson, Hiroshi Tsurumaru, Alfred Wiedensohler, Cerina Wittbom, John A. Ogren, Atsushi Matsuki, Seong Soo Yum, Cathrine Lund Myhre, Ken Carslaw, Frank Stratmann, Martin Gysel

**Affiliations:** 1 Laboratory of Atmospheric Chemistry, Paul Scherrer Institute, Villigen 5232, Switzerland; 2 Experimental Aerosol & Cloud Microphysics, Leibniz Institute for Tropospheric Research, Permoserstrasse 15, Leipzig 04318, Germany; 3 Netherlands Organisation for Applied Scientific Research, Princetonlaan 6, Utrecht 3584, The Netherlands; 4 Department of Physics, University of Helsinki, Gustaf Hällströmin katu 2, Helsinki 00014, Finland; 5 Hyytiälä Forestry Field Station, Hyytiäläntie 124, Korkeakoski 35500, Finland; 6 Laboratoire de Météorologie Physique, 4 Avenue Blaise Pascal, Aubiere, Cedex 63178, France; 7 School of Physics and CCAPS, National University of Ireland Galway, University Road, Galway, Ireland; 8 ECPL, Department of Chemistry, University of Crete, Voutes, Heraklion 71003, Greece; 9 IERSD, National Observatory of Athens, P. Penteli, Athens 15236, Greece; 10 Cooperative Institute for Research in the Environmental Sciences, University of Colorado, Boulder, Colorado 80309, USA; 11 Department of Atmospheric Science, Yonsei University, Seoul 03722, South Korea; 12 Institute for Marine and Atmospheric Research, University of Utrecht, Utrecht 3508 TC, The Netherlands; 13 Institute for Energy and Climate Research (IEK-8): Troposphere, Forschungszentrum Jülich, Jülich 52425, Germany; 14 Department of Physics, Lund University, Lund 221 00, Sweden; 15 Institute of Nature and Environmental Technology, Kanazawa University, Kakuma-machi, Kanazawa 920-1192, Japan; 16 Faculty of Science Division I, Department of Physics, Tokyo University of Science, 1-3 Kagurazaka, Shinjuku-ku, Tokyo 162-8601, Japan; 17 School of Earth and Environment, University of Leeds, Leeds LS2 9JT, UK; 18 Federal Environment Agency, Corrensplatz 1, Berlin 14195, Germany; 19 NILU -Norwegian Institute for Air Research, Instituttveien 18, Kjeller 2007, Norway; 20 Energy Research Center of the Netherlands, Petten 1755 ZG, The Netherlands; 21 Grolimund+Partner AG, Thunstrasse 101a, Bern 3006, Switzerland; 22 School of Chemical & Biomolecular Engineering, Georgia Institute of Technology, Atlanta, Georgia 30332, USA; 23 Foundation for Research and Technology - Hellas, Heraklion, Crete GR 700 13, Greece; 24 School of Earth and Atmospheric Sciences, Georgia Institute of Technology, Atlanta GA 30332, USA; 25 Institute for Atmospheric and Climate Science, Federal Institute of Technology, Universitätsstrasse 16, Zurich 8092, Switzerland

**Keywords:** Atmospheric chemistry, Attribution

## Abstract

Cloud condensation nuclei (CCN) number concentrations alongside with submicrometer particle number size distributions and particle chemical composition have been measured at atmospheric observatories of the Aerosols, Clouds, and Trace gases Research InfraStructure (ACTRIS) as well as other international sites over multiple years. Here, harmonized data records from 11 observatories are summarized, spanning 98,677 instrument hours for CCN data, 157,880 for particle number size distributions, and 70,817 for chemical composition data. The observatories represent nine different environments, e.g., Arctic, Atlantic, Pacific and Mediterranean maritime, boreal forest, or high alpine atmospheric conditions. This is a unique collection of aerosol particle properties most relevant for studying aerosol-cloud interactions which constitute the largest uncertainty in anthropogenic radiative forcing of the climate. The dataset is appropriate for comprehensive aerosol characterization (e.g., closure studies of CCN), model-measurement intercomparison and satellite retrieval method evaluation, among others. Data have been acquired and processed following international recommendations for quality assurance and have undergone multiple stages of quality assessment.

## Background & Summary

Cloud condensation nuclei (CCN) are the subset of aerosol particles able to form cloud droplets. They influence cloud microstructure and precipitation processes, which in turn affect the radiative properties of clouds, atmospheric circulation and thermodynamics, as well as radiative budgets^1^. This has implications at various scales. In terms of radiative forcing, aerosol-cloud interactions are the least understood anthropogenic influence on climate^2^. The uncertainty in aerosol-induced radiative forcing of ±0.70 W m^−2^ (from a mean of −0.55 W m^−2^) is twice the uncertainty for CO_2_ (±0.35, mean +1.68 W m^−2^). At the regional scale, aerosol-cloud interactions can change radiation and precipitation processes^2,3^. Reducing the large uncertainty in aerosol effects is a major challenge in increasing confidence in global and regional climate change projections^2,4,5^.

Key to uncertainty reduction is a better understanding of both large-scale/long-term and regional scale/short-term aerosol properties including their number concentration, number size distribution, chemical composition and ability to form cloud droplets. While proxies of these variables are observed by satellites, the resolution is too coarse for studying aerosol-cloud interactions, making numerous in-situ measurements necessary^1,6^. Many short-term datasets from intensive field measurements are available^7–10^ that enhance our process understanding but often provide a patchy and skewed picture of aerosol characteristics as such efforts are designed to explore particular ambient conditions at varying locations. Collocated long-term observations of CCN activity, particle number size distribution and chemical composition are sparse, especially in the vertical dimension^8,11,12^. To evaluate models’ performances against measurements in order to improve climate projections, observationally derived long-term regionally representative aerosol properties are indispensable.

Quality assured long-term and regionally-representative datasets acquisition requires that the following criteria be met: (i) an infrastructure consisting of several observing locations that are representative of a variety of environments; (ii) harmonized aerosol measurement techniques following standard operation procedures, and ideally with instruments regularly calibrated at certified calibration centers for quality assurance; (iii) harmonized quality assessment for data; and, (iv) concurrent measurements of sufficient types of aerosol properties to resolve aerosol-cloud processes. Particle number concentrations alone do not constrain cloud condensation nuclei concentrations because unresolved variations in their size, hygroscopicity and mixing state (which is a function of their chemical composition) introduce important uncertainty in predicted CCN number concentration^13,14^. It is the combination of measured variables that makes datasets useful to study aerosol-cloud interactions.

At the European level, the Aerosols, Clouds, and Trace gases Research InfraStructure (ACTRIS), among other objectives, aims at increasing the ‘availability of long-term observational data relevant to climate and air quality research on the regional scale produced with standardized or comparable procedures’ (http://www.actris.eu/). ACTRIS’ particular focus on the comprehensive characterization of aerosol particles makes it the largest network of long-term ground based stations of collocated observations of CCN, particle number size distributions and online particle chemical composition. Complementary networks, such as the Atmospheric Radiation Measurement Program (ARM, http://dis.arm.gov/sites), the World Meteorological Organization’s Global Atmosphere Watch (WMO-GAW, https://gawsis.meteoswiss.ch/GAWSIS//index.html#/), and individual initiatives are covering other regions of the world.

Here, we present a harmonized dataset of CCN number concentrations and particle number size distributions for 11 stations, and particle chemical composition for a subset of these stations. Available data have been collected from observatories globally. Criteria were a) the use of a Droplet Measurement Technologies Continuous-Flow Streamwise Thermal Gradient Chamber, also CCN counter (CCNC), a validated type of mobility particle size spectrometer (MPSS), and an Aerodyne Research Inc. aerosol mass spectrometer (AMS) or aerosol chemical speciation monitor (ACSM); b) data quality (see Methods and Technical Validation); and c) data covering at least 75 % of all seasons of one year. Among these stations are eight ACTRIS (GAW) sites, one Earth System Research Laboratory site of the US National Oceanic and Atmospheric Administration, and two Asian initiatives. The entire data record spans 98,677 instrument hours for CCN data, 157,880 for particle size distribution and 70,817 for chemical composition, and represents nine different environments. The general instrumental set-up and location-related specifics are described in the Methods section. The records are available as Network Common Data Form (NetCDF) files (Data Citation 1) whereby each file contains data from one instrument (CCNC, MPSS or AMS) and station. These NetCDF records are additionally available through the ACTRIS data portal (http://actris.nilu.no/Content/Products). The harmonized multi-year data of three key variables (cloud condensation nuclei, particle number size distribution and chemical composition) for aerosol-cloud interactions is a unique collection to test satellite retrieval methods and to evaluate global climate models targeting the reduction of the related uncertainty in radiative forcing.

## Methods

This section provides an overview of the general experimental design followed by descriptions of the individual measurement sites and instruments. Where applicable, discussions of site-related specificities are included. Information on recommended operation procedures for data quality assurance for all three aerosol instrument types is provided in more detail since standard operation procedures are currently being developed or have only recently been established. Additionally, we report site-specific instrumental calibration and particle loss calculations where applicable.

### General study design

Table 1 provides an overview of stations with their names, geographical location, available data files and measurement protocols.

**Figure 1:**
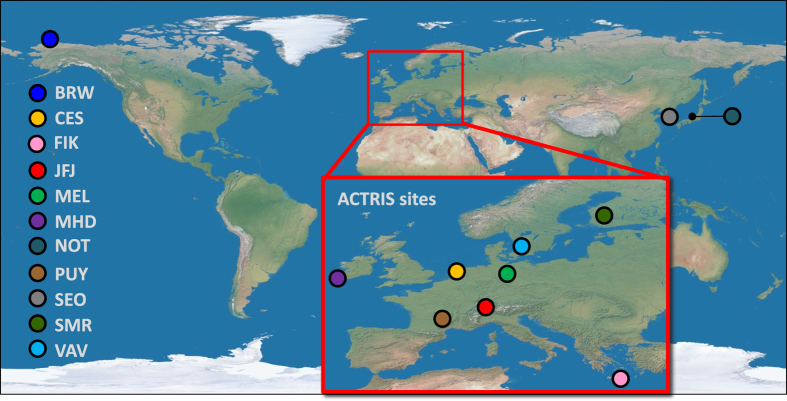
Map of sampling sites included in the dataset. Made with Natural Earth III (http://www.shadedrelief.com/natural3/pages/textures.html).

**Figure 2:**
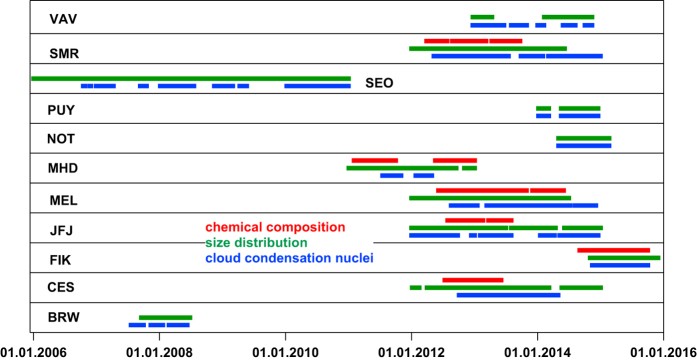
Time coverage of the record of harmonized data. More data are available through the data portal EBAS (http://ebas.nilu.no).

A prerequisite to be considered in this work were parallel and standard measurements of cloud condensation nuclei concentrations and aerosol number size distributions covering at least 75 % of one year. These data (Data Citation 1) were provided from 11 stations (see Table 1, Figs 1,2). At six of these stations, additional standard aerosol chemical composition data were available (see Table 1). Stations include eight shared ACTRIS/GAW sites (marked with an asterisk in Table 1) in Europe, two in Asia and one in North America. The represented environments include continental background, rural background, urban, coastal, boreal forest, Arctic, Mediterranean and high altitude conditions. At most stations more data of one or two variables are available, however, those time periods are not included here if CCN data were not available.

**Figure 3:**
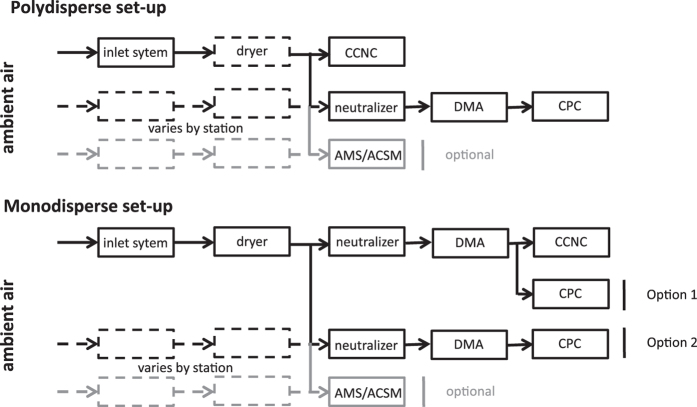
General measurement set-up. The upper panel shows the set-up for polydisperse cloud condensation nuclei (CCN) measurements. Depending on the station specific set-up, the same or different inlet systems were used for the size distribution and optional chemical composition measurements. Dashed boxes and lines indicate that the specific set-up varied by station. The lower panel shows the same for monodisperse CCN measurements, whereby size distributions were either measured after the same neutralizer and differential mobility analyzer (DMA) or behind a second system (indicated with option 1 and 2). The methods section specifies which set-up each station used. ACSM=aerosol chemical speciation monitor, AMS=aerosol mass spectrometer, CCNC=cloud condensation nuclei counter, CPC=condensation particle counter.

Figure 3 presents the general instrumental set-up with cloud condensation nuclei counters and mobility particle size spectrometer measurements for the poly- and monodisperse operation alternatives, and the optional chemical composition measurement. In the case of polydisperse CCN measurements, the aerosol is sampled and all sizes are simultaneously measured by the CCNC. In the monodisperse case, a size selection prior to the CCN analysis occurs. While different information can be gained by the two measurement options, this aggregated dataset considers only the time series of CCN number concentrations at various supersaturations (SS, see Table 2). Time series are also provided for the submicrometer aerosol number size distribution and, where available, chemical species including particulate sulfate, nitrate, ammonium, organics, chloride and sea salt. In some cases, all three types of instruments shared the same aerosol inlet, while at other stations, separate, yet closely positioned, inlets were used.

All data originators (instrument principle investigators) submitted data in their preferred format (Nasa Ames 1001 from EBAS or instrument specific format) to this effort from which the first author constructed the time series and converted all data to standard temperature and pressure (STP) where necessary. Temperature and pressure data were either available from the data originators or in the EBAS data base (http://ebas.nilu.no). Thereafter, all data types were averaged to the full hour with the time stamp being the end of the measurement interval and the time series were converted to UTC. In case of the Puy de Dôme observatory (PUY), averages are over 4 h due to longer CCN monodisperse scan times. Further data treatment associated with quality assurance is explained in the ‘Technical Validation’ section. Hereafter, this dataset is called ‘aggregated’ or ‘secondary’ dataset. Several of the primary measurement datasets, this is without the processing and aggregation by the first author, were archived in and are available from the EBAS data base. EBAS is the primary data repository for all ACTRIS near-surface data, also hosting GAW-World Data Center for Aerosols (GAW-WDCA, http://www.gaw-wdca.org) and all European Monitoring and Evaluation Programme (EMEP) data amongst others.

### Site description and inlet systems

This section includes details about the measurement stations’ characteristics and regional representativeness, together with specificities of the aerosol inlet systems. Generally, all inlet systems comply with the WMO-GAW aerosol and ACTRIS standards and recommendations. The basic rules of aerosol sampling include^15^:

- in case of cloud presence at the station, keeping droplets either from entering or evaporating them to sample the residuals;
- keeping diffusional and inertial particles losses as well as evaporation of volatile particulate components to a minimum;
- ensuring relative humidity <40 % upstream of the instruments in the sample line. At RH <40 %, the particle diameter will change less than 10 % as compared to fully dry particles, and thus will not introduce biases for size determination or change other characteristics. Furthermore, no condensation of water vapor in the inlet system will occur which could impair instrument performance.

Most stations have been audited by the World Calibration Center for Aerosol Physics (WCCAP) and pictures of some stations can be found here: https://www.tropos.de/forschung/grossprojekte-infrastruktur-technologie/technologie-am-tropos/qualitaetssicherung-von-aerosolmessungen/.

### Barrow (BRW)

The Barrow facility is operated under the Earth System Research Laboratory of the US National Oceanic and Atmospheric Administration and located near the Arctic Ocean. The station is surrounded by flat tundra, large lagoons, and lakes. The predominant wind direction is from east-northeast from the Beaufort Sea with minimal anthropogenic pollution. Generally, the station’s environment can be described as Arctic maritime climate affected by variations of weather and sea ice conditions in the Central Arctic (see also https://gawsis.meteoswiss.ch/GAWSIS//index.html#/search/station/stationReportDetails/489).

The aerosol inlet is a standard NOAA site system as described by ref. 16. The inlet consists of a roughly 10 m high intake stack with 21.4 cm inner diameter with a flow of 1000 l min^−1^. The sample flow does not require specific drying due to the temperature difference between the ambient air and laboratory environment. Typically, the relative humidity is <30 %. From the center part of the stack, the aerosol sample flow is extracted and isokinetically split to the various instruments. Each stainless steel line has a controlled flow of 3 l min^−1^ to avoid particle losses. The inlet system is built in such a way that losses of 10 μm particles are <10 % and <5 % for particles between 0.01 and 1 μm.

### Cabauw (CES)

The Cabauw Experimental Site for Atmospheric Research (CESAR) is operated by the Royal Netherlands Meteorological Institute (KNMI) and located about 40 km from the North Sea at 0.7 m below sea level. The station’s environment is typical for north-west Europe and can be described as background continental and maritime. Influences from the cities of Utrecht and Rotterdam (20 and 30 km, respectively) cannot be excluded^8^, as well as from agricultural activities of grassland management and animal keepings^17^.

Aerosol is sampled at the 60 m mark from the 220 m high CESAR tower. The inlet system consists of the following sections: 1) four PM_10_ sampling heads, 2) two Nafion dryers (type PD-200T) to keep the sample flow below 40 % RH, 3) a 60 m stainless steel tube with a 66.8 l min^−1^ laminar flow, and 4) a manifold to serve the various instruments with sample air^18^. Particle losses have been evaluated taking into account the calculations and measurements as provided in refs 17,19.

### Finokalia (FIK)

The Finokalia station (http://finokalia.chemistry.uoc.gr/) is operated by the University of Crete on the northern coast of the island of Crete. It is located at the top of a hill and representative of maritime background conditions as the nearest city, Heraklion, is about 70 km away. Two seasons can be distinguished. The dry season from April to September is characterized by elevated wind speeds from the north-northwest. In the wet season from October to April in addition to the north-northwesterly winds influence from the south-southwest (Sahara) becomes important^20^. Aged aerosol populations from the marine boundary layer, continental Europe, the Saharan desert, and summer biomass burning are frequently observed.

Aerosol measurements are conducted in a dedicated building at the station equipped with various aerosol inlets which are situated at 4 m above ground level. Generally, the systems consist of a sampling head (total aerosol, PM_10_, PM_2.5_ or PM_1_), a short stainless steel tube with a laminar sample flow, and dryers to keep RH below 40%.

Note that the station abbreviation ‘FIK’ is used in the GAW system, while ‘FKL’ is used in ACTRIS and other protocols and ‘GR0002R’ in the EMEP database.

### Jungfraujoch (JFJ)

The high-alpine research station Jungfraujoch is located in the Swiss Alps at 3580 m above sea level on a ridge between two mountains higher than 4000 m. Aerosol measurements are conducted by the Paul Scherrer Institute’s Laboratory of Atmospheric Chemistry. The station is considered a continental background site since it is far away from major anthropogenic emission sources. Aerosol properties exhibit a strong seasonal cycle with lower concentrations in winter when free tropospheric conditions prevail. During the warm season, concentrations are higher owing to injections of more polluted boundary layer air masses due to thermal convection. A more detailed description can be found in ref. 21. Additionally, tourism related emissions can influence aerosol measurements particularly in the summer months and during favorable weather conditions year-round. Such local influences of pollution have been removed from the dataset by visual inspection to make the dataset representative of the regional background conditions^22^.

In the Sphinx laboratory on Jungfraujoch, the heated (~20 °C) inlet collects all aerosol particles and cloud droplets up to 40 μm. As the cold sample flow (July mean temperature is around −1 °C) enters the laboratory which is kept at approximately 25 °C, all condensed water evaporates, hence interstitial as well as activated aerosol particles are measured. Losses for the whole inlet system are below 5% for particles between 10 and 750 nm diameters. Inside the laboratory, the dried aerosol sample is distributed to a series of instruments including the permanently installed CCNC and scanning mobility particle sizer (SMPS), as well as to the time-of-flight aerosol chemical speciation monitor (ToF-ACSM) for the respective measurement period. More detailed descriptions of the inlet can be found in refs 11,22,23.

### Mace Head (MHD)

Mace Head is located on the west coast of Ireland roughly 100 m from the Atlantic shoreline. It is operated by the National University of Ireland, Galway, and is a GAW, EMEP and ACTRIS station. Aerosol populations represent north-east Atlantic background conditions. The closest city, Galway, is about 90 km away. Sixty four percent of the time, air arriving at Mace Head is either clean or pristine marine air, the remainder being polluted to different degrees, either from local sources (modified marine air masses) or long-range transport from the UK and continental Europe^24^.

Aerosol measurements are conducted from a shore laboratory using the 10 m high stainless steel community sampling duct with a diameter of 10 cm and operated at 150 l min^−1^. The sample air is dried to <40 % RH. The 50 % upper size cut-off for the carrier duct is at 10 μm, and losses of particles <1 μm are <5 % for low and reach 30 % for 15 m s^−1^ wind speeds^25^.

### Melpitz (MEL)

The Melpitz research station is operated by the Institute for Tropospheric Research, Leipzig, which also hosts the World Calibration Center for Aerosol Physics. The site, located 45 km north-east from Leipzig, is representative of more anthropogenically influenced Central European background conditions^26^. It is surrounded by flat grass lands, fields and forests. Westerly wind conditions bring air masses from the Atlantic with lower aerosol particle mass concentrations, while easterly winds transport continental air masses with more accumulated particle mass concentration^27^.

Online aerosol instruments are located in the container laboratory and sample from an inlet 6 m above ground which carries a PM_10_ head followed by an aerosol diffusion dryer that maintains RH below 30 % (ref. 28). Particle transmission is near 100 % for the size range from 20 to 800 nm (ref. 28).

### Noto (NOT)

The Noto Ground-based Research Observatory is located at the eastern tip of the Noto Peninsula at the west coast of Japan. The peninsula reaches about 150 km into the sea and the nearest provincial cities are Toyama and Kanazawa, 85 and 115 km away, respectively. The remote location allows for monitoring of atmospheric background conditions in East Asia as well as long-range transported pollution originating from continental East Asia^29^.

The aerosol inlet system draws air from 14.7 m above ground at a flow rate of 78 l min^−1^ through a stainless steel tube (5.65 cm outer diameter). An isokinetic flow splitter distributes the sample air to the individual instruments where the air is dried before analysis^29^.

### Puy de Dôme (PUY)

Puy de Dôme is a mountain station at 1465 m altitude in the French Massif Central. Aerosol measurements are conducted by the Laboratoire de Météorologie Physique. The station is surrounded by forests and agricultural land and the influence of the nearest city, Clermont-Ferrand, 396 m a.s.l., 16 km to the east, is limited, especially during night time. The area is accessible by train (electrically driven) which stops some 500 m away, which has mostly a negligible influence on the aerosol measurements^30,31^. The predominant wind direction is from the west, while the moderate altitude of the station enables characterization of the planetary boundary layer, lower free troposphere, nocturnal residual layer and their interfaces. The aerosol concentration is lowest during wintertime, when the influence of the free troposphere is highest^30^.

As the site is covered in clouds 50% of the time during winter, aerosol is sampled from a whole air inlet (WAI) that efficiently samples particles and droplets <35 μm at wind speeds <8 m s^−1^. Due to the temperature difference between the ambient air and the laboratory, the sample air is not actively dried and it is typically <40% RH. After water vapor dissipation the aerosol population is thought to represent an aerosol after the natural dissipation of a cloud. Losses are <5% for particles larger than 15 nm (ref. 30). Particle number concentrations and size distributions are measured downstream of the same WAI.

### Seoul (SEO)

Seoul is a megacity in South Korea with more than 10 million inhabitants. Measurements were conducted by the Yonsei University on their campus which is located in the northwestern part of the city. More precisely, the instrumentation was located on the sixth floor of a building roughly 300 m away from the nearest main traffic roads. The site can be characterized as urban background. It is also characteristic of seasonal differences due to the summer monsoon^32^.

The sample air to the instruments was neither dried nor diluted. The length of the inlet lines to each instrument was about 1 m built with 0.25 inch conductive tubing. Particle losses were minimal for the submicrometer size range.

### Hyytiälä (SMR)

The Station for Measuring Ecosystem- Atmosphere Relations (SMEAR II) is located in the Hyytiälä Forestry Field Station, in southern Finland, and is operated by the University of Helsinki. It is surrounded by boreal coniferous forest, dominated by scots pine, and is representative of the boreal environment^33^. The nearest larger city, Tampere, is located 60 km to the south-west. Air masses at SMR originate from the Arctic and Europe, but aerosol concentrations are typically low^8^. Local pollution sources are of minor impact but non-negligible, as there are e.g., sawmills, light traffic, minor agriculture and houses nearby. Local pollution is usually easily distinguished by aerosol plumes in the data.

Cloud condensation nuclei number and particle number size distribution measurements are conducted from a PM_10_ inlet 8 m above ground. The inlet flow is 150 l min^−1^, and it is dried to RH <40% before the flow is split to the individual instruments. Particle losses in the inlet system are minimal.

### Vavihill (VAV)

Vavihill station, operated by Lund University, is located in southern Sweden and is surrounded by grasslands and deciduous forest. It serves as a continental background station appropriate to study continental European pollution outflow to the North. South-westerly winds are dominant^34^. The nearest village is 10 km away, while the largest cities are located in the west to south-east sector (Helsingborg 25 km away, Lund 46 km, Malmö and Copenhagen 60–70 km).

Aerosol is sampled through two standard PM_10_ inlet heads^35^ through a stainless steel tube roughly reaching 2 m above the laboratory container. For the inlet connected to the particle number size distribution measurements, the air is dried to RH <40%, whereas after the second inlet for the polydisperse CCN measurements, no drier is used.

### Instrument descriptions

Here we describe the measurement principles, major uncertainties and standard operation procedures for the cloud condensation nuclei counter, the particle size spectrometers and the aerosol mass spectrometers. Each general instrument description is succeeded by more detailed information for each site.

### Cloud condensation nuclei counter (CCNC)

All stations used the only commercially available CCNC, model CCN-100 from Droplet Measurement Technologies (DMT, Boulder, USA), which is a Continuous-Flow Streamwise Thermal Gradient Chamber, described in detail in refs 36,37. Instrument modifications are discussed under the station headings below if applicable.

The CCNC consists of a cylindrical continuous-flow chamber in which aerosol can be exposed to a constant (user-defined) supersaturation as follows. An aerosol sample flow is guided through the center of the cylinder by a particle-free laminar sheath flow. Particles that activate at a critical supersaturation lower than the set supersaturation form droplets. The size distribution of droplets (particles with diameter larger than 1 μm) exiting the activation column after a roughly 10 s exposure to supersaturation is counted by an optical particle counter. The centerline supersaturation is generated by applying a controlled (and constant) streamwise temperature gradient at the cylinder wall; by maintaining the inner wall wet, heat and water vapor continuously diffuse towards the center of the tube. Because water vapor has a lower molecular weight than moist air, diffusion of water vapor is faster than heat and the centerline becomes supersaturated^37^. A constant flow rate, chamber pressure and streamwise temperature gradient ensure a quasi-constant supersaturation for the developed region of the flow in the chamber^36^.

Refs 36,38,39 provide recommendations for the operation of the CCNC. There are two main operation modes as shown in Fig. 3. Polydisperse aerosol activation is measured when simply sampling ambient air, while in the monodisperse operation mode, particles are size selected by means of a differential mobility analyzer (DMA) prior to entering the CCNC.

Table 2 indicates in which mode the instruments were operated. In both cases time series with total number concentrations of CCN at a certain supersaturation can be derived, if the scanned diameter range in the monodisperse measurements has a sufficiently large upper detection limit such that only a very minor fraction of droplet activating particles is not captured. This was the case for all stations with a monodisperse measurement set-up. Therefore there is no difference in the data files presented here except for a lower time resolution in the case of the PUY station. Independent of the operation mode, the common supersaturations recommended to be measured are 0.1, 0.2, 0.3, 0.5 and 1.0%.

Table 2 indicates the actually measured supersaturations. At each station, at least one of these values was measured. The supersaturation of 1.0% is recommended to compare CCN with the total number concentration for quality assurance. See section ‘Technical Validation’ for details. Further recommendations include:

- flow rates settings a) to avoid too long residence times in the lines upstream of the activation column to avoid diffusion losses, or too short residence times in the column that can limit droplet growth; b) to guarantee laminar flow conditions by setting the correct aerosol to sheath flow ratio (1:10).
- setting temperatures in the correct ranges and time intervals to ensure stable supersaturation values and reliable counting statistics, as well as stepping from the highest to the lowest supersaturation.

Main factors that introduce uncertainty in the measured CCN number concentrations are the flow rate and the calibration of the instrument’s supersaturation, and changes in pressure e.g., during airborne operations^40^. The flow rate has a direct impact on the supersaturation and together with the sheath flow is a parameter to convert counts to a number concentration. Therefore ref. 38 recommends calibrating the flow rate at least every six months. With respect to the supersaturation calibration, details are given in the above mentioned references for ammonium sulfate as well as for sodium chloride in ref. 41. Briefly, ammonium sulfate particles (preferred over sodium chloride) of a selected size are introduced into a condensation particle counter (CPC) and the CCNC in parallel while supersaturations are stepped (‘S-scan’). This yields the temperature gradient needed to activate particles with the given diameter. The temperature gradient determines the supersaturation at a given flow rate. Alternatively, the diameter of the particles can be scanned or stepped at a fixed supersaturation (‘D-scan’). Here, the critical diameter is derived from the given temperature gradient (supersaturation). Based on the thermodynamic properties of ammonium sulfate particles the critical supersaturation *SS*_*crit*_ corresponding to *D*_*crit*_ or *D*_*set*_, and temperature can be determined. This can be done by applying the Aerosol Diameter Dependent Equilibrium Model (ADDEM, ref. 42), the Aerosol Inorganic Model (AIM) like in ref. 41, the Pitzer-interaction model^43^ or a specifically prepared lookup-table (Supplementary Material 1) which is based on an implementation of the Pitzer-interaction model^44^ and cross-validated against ADDEM. Calibration curves of the temperature gradient in the CCNC activation column versus the derived supersaturation are then created based on which the uncertainty in the determination of the supersaturation can be calculated. Generally, the target accuracy for *SS*>0.2% is ±10%, in relative terms, and *ΔSS*≤0.03%, in absolute terms.

During measurements, the actual supersaturation can deviate from the target setting. For such instances, the recommendation^38^ is to linearly interpolate to the target supersaturation for deviations <20%. This was applied to this dataset. For larger deviations, data is reported as missing. Importantly, supersaturation calibrations need to be carried out at the flow rate and pressure level at which the instrument will be operated. Ref. 38 provides information on data processing procedures for how to include temperature readings and treatment of diffusion losses, and, for monodisperse measurements specifically, the correction for multiply-charged particles and inversion routines (see paragraph on particle size spectrometers).

In case of high CCN number concentrations (>5000 cm^−3^), the supersaturation and droplet sizes can decrease, because of the higher water vapor depletion^45^. This can affect the derived CCN number concentration. This dataset has not been corrected for this potential effect.

Reliability and comparability of the datasets presented here were ensured by the application of the ACTRIS technical standards as described above (if not described otherwise below). In the case of the non-ACTRIS stations and earlier measurements, the same procedures were followed nevertheless as they had been established as ‘good practice’ within the community beforehand. For each station’s CCN measurements, Table 2 provides details on the data treatment protocol which includes information on the deployed instrument model, its operation mode, and acquisition and data processing software used to create the data record. References describing data acquisition and processing following the protocols are included. Protocols for CCN datasets are named ‘P_CCNC_nameofstation’.

### Station specific CCNC remarks

#### BRW

The instrument had been calibrated at a lower pressure level (840 hPa) than the operational level (sea level). Therefore the uncertainty in the supersaturation determination is between 10 and 20%.

#### CES

Specific particle losses due to the inlet line from the manifold and within the instrument have been calculated. Transmission of particles starting at the size range expected to activate at 1% supersaturation (roughly 50 nm) is >90%. The losses have been taken into account.

#### FIK

At Finokalia, the CCNC is connected to a PM_1_ head. The instrument is calibrated regularly with sodium chloride particles. Differences to the ACTRIS SOP calibration with ammonium sulfate are described in detail in ref. 43.

#### JFJ

**Figure 4:**
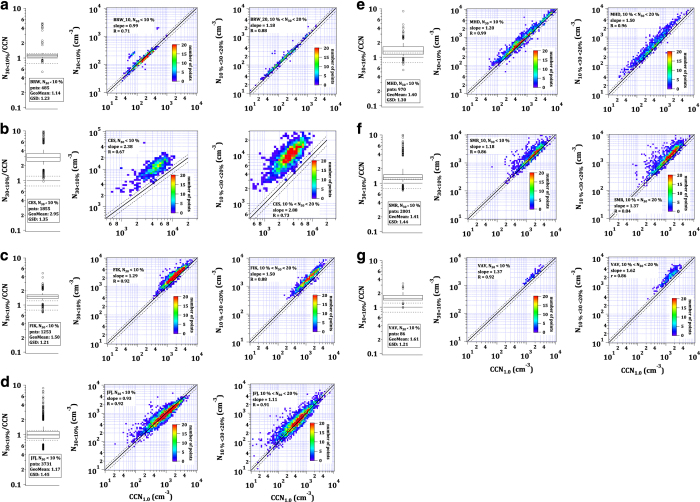
Data quality check for polydisperse measurements at 1% supersaturation. Each panel (**a–g**) shows for each station, in alphabetical order, the ratio of the total particle number to CCN_1.0_ when the number of particles <30 nm (N_30_) makes up between 10 and 20 % (right) and <10% (middle) of the total particle number. Results are presented in logarithmic bins where the color code shows the number of points per bin. The slopes and correlation coefficients of the curves are provided. The black line denotes the 1:1 line and the dashed black lines indicate the range of expected uncertainty from particle counting. The left panels show box and whiskers plots of the ratio N_30<10%_/CCN_1.0_ with the median, interquartile range, 10th and 90th percentiles, and points beyond the 2.5th and 97.5th percentile. The grey solid lines and dashed lines indicate the same as black lines in the right hand side plots. The number of total points is provided (*pnts*) as well as the geometric mean (*GeoMean*) and geometric standard deviation (*GSD*).

The CCNC measured behind the above described aerosol inlet with a total flow of 1 l min^−1^ until February 2013, and thereafter with a flow of 0.75 l min^−1^. The performance of the CCNC varied throughout the measurement period. In 2012 the instrument ran comparably to conditions reported in ref. 11 with a maximum uncertainty of reported supersaturations of 10%. In 2013, after exchanging the Nafion membrane, the instrument calibration curves varied more strongly throughout the year leading to a maximum uncertainty of 16%. In 2014, the maximum uncertainty was 4%. A comparison of CCN data at 1% supersaturation with the integrated SMPS particle number concentration showed that the CCN concentration in 2012 was underestimated by about 40% while it was overestimated by about 30% in 2013 after the membrane exchange. Data for these two years were corrected accordingly (see Fig. 4).

#### MEL

A DMA is used to perform size-segregated CCN measurements. The aerosol to sheath air flow rate is kept at 1:10. Multiply-charged particles cannot be avoided in the selection process, hence CCN data is corrected by applying the bipolar charge distribution^46^. To quality check CCN data, particle number size distributions from a separate dual mobility particle size spectrometer (described below) have been used.

#### NOT

Monodisperse CCN analysis was performed following the Scanning Mobility CCN Analysis (SMCA) system^29,47^. The sample air was dried with two silica gel diffusion dryers. Subsequently particles were selected by their mobility diameter with a DMA. The monodisperse aerosol sample flow was split between a CPC and the CCNC for measurements of total particle and CCN number size distributions. The CCNC was calibrated regularly with ammonium sulfate following a procedure comparable to the ACTRIS SOP. Data analysis was conducted with the SMCA software package^47^ which includes multiple-charge correction for particle and CCN number size distributions.

#### PUY

Different from all other stations, at PUY a miniature version of the DMT CCNC-100 is operated^48^ at only one supersaturation (0.2%) in the monodisperse mode. The aerosol flow is 0.015 l min^−1^ adjusted with a sheath flow to 0.1 l min^−1^ total. To reduce particle losses a bypass flow of 0.4 l min^−1^ is added. Standard calibration and operation procedures do not differ from the ACTRIS SOP.

#### SEO

Since measurements were conducted before the ACTRIS projects and outside of Europe, calibration and operation of the CCNC were based on the methods described in ref. 41. Those methods constitute a major reference to the current ACTRIS recommendations.

#### SMR

In addition to the polydisperse CCN measurements described in this study, size-resolved CCN number concentration measurements were performed concurrently since 2007. Those measurements are described in detail in ref. 49 and are available from the EBAS data base.

There are no specific remarks for the stations MHD and VAV.

### Mobility particle size spectrometers

The mobility particle size spectrometer, often called scanning mobility particle sizer (SMPS) or the similar differential mobility particle sizer (DMPS), measures the number size distribution of submicrometer particles by counting particles of the different sizes that are selected based on their electrical mobility. Commonly, an MPSS consists of an impactor, a bipolar diffusion charger (often called neutralizer), a DMA, and a CPC setup in series. The bipolar diffusion charger brings the particles into an approximate bipolar charge equilibrium. The DMA is a cylinder with a charged electrode at its center. At the outer side the aerosol sample enters and is then guided to the bottom in a laminar flow by particle free sheath air. By applying a voltage between outer cylinder and electrode, all charged particles move towards the center rod and only particles of a certain size move directly towards the exit of the column and are counted. The upstream impactor removes particles larger than the upper DMA size limit, which enables correction for larger multiply charged particles with the same mobility diameter as singly charged smaller particles. The scanning of different voltages in the DMA results in an electrical mobility distribution. This can be transformed into particle number size distributions by using an inversion method. All inversion methods applied here have been tested and yield reliable results^50^. A DMA transfer function is included in the inversion calculation to account for the transmission of particles through the DMA at the given flow rate and particle size. Additionally, diffusion losses in the whole system (including the neutralizer) are considered via the ‘effective length’, as well as the counting efficiency of the CPC in particular with respect to their lower cut-off diameters^50^.

The reliability of the measurements is subject to a number of operating parameters as summarized below based on refs 39,50:

- flow rates: A 1% error in the sheath air flow rate result in a shift of 1% in selected the particle mobility. At the typical ratio of aerosol to sheath air flow 1:10, a leak in the sheath air flow of 1% results in a 10% error in the aerosol flow which directly impairs the particle number concentration.
- Leaks and contamination of the flows result in large errors that are difficult to quantify.
- The mobility of the particle depends on the actual temperature and pressure at which the system is operated. This aspect is important for e.g., mountain stations. Pressure changes of 30 hPa result in a 1% error in sizing.
- The relative humidity of the aerosol as well the sheath air flow should be kept <40% to avoid hygroscopic growth of particles which would change their diameter.

MPSSs are calibrated by verifying the sizing with polystyrene latex (PSL) spheres of a given size. The instrument should determine the particle size within the PSL size uncertainty of 2.5% and the tolerable variance in the sheath air flow rate of 1%. This implies that flow rates were regularly if not continuously checked. In addition, zero-checks were conducted to avoid false counts.

Details on each station’s mobility particle size spectrometer are given in Table 3. Different from the CCNC, there are several commercial models available and some user groups operate their own custom-built versions. Due to the many different models as well as the sensitivity of the measurements to a number of operational parameters as outlined above, comparability of results can be hampered. For this reason it is particularly important to ensure compliance with the standard technical requirements, operation procedures and the use of validated inversion routines for data analysis as provided by ref. 50 which serves as guideline within ACTRIS and WMO-GAW among others. All stations followed these guidelines including non-ACTRIS sites, if not stated otherwise. In Table 3, datasets from station operators that participated in the intercomparison described in ref. 50 are marked with ‘*’. In addition to their instruments, also inversion routines have been tested.

Despite compliance with the protocols, deviation of results between instruments is expected. In a number of intercomparison workshops at the WCCAP (ref. 50) it was found that uncertainties within 10% can be expected for particles in the size range 20 to 200 nm, while deviations become significantly larger for smaller particles. Also at the higher end, divergence was observed. This should be kept in mind when interpreting particle size distributions and size resolved number concentrations.

Table 3 provides details on the size distribution data treatment protocol which includes information on the deployed instrument model, its operation mode, and acquisition and data processing software used to create the data record. References describing data acquisition and processing following the protocols are included. Protocols for size distribution datasets are named ‘P_size_nameofstation’.

### Site specific remarks

#### JFJ

The JFJ SMPS is connected to the total air aerosol inlet and operates with a sample flow of 0.3 l min^−1^ and a sheath flow of 3 l min^−1^. To validate the integrated particle number concentration as derived from the SMPS measurements, in addition to participating in intercomparisons at the WCCAP (ref. 50), it is compared to the number concentration determined by the CPC for periods in which very few particles under 20 nm are present. Particles larger than 600 nm play only a minor role in the number concentration at the measurement site. A size-independent, time-dependent correction factor is determined and applied. Periods with over 20% discrepancy are removed from further data analysis^22^.

#### SEO

As measurements were taken outside the ACTRIS geographical reach, the instrument did not participate in intercomparison activities. Details of its operation are provided in ref. 32.

There are no specific remarks for the stations BRW, CES, FIK, MEL, MHD, NOT, PUY, SMR, and VAV.

### Aerosol mass spectrometers

A variety of aerosol mass spectrometers has been developed within the past 15 years by Aerodyne Research Inc. (ARI, Billerica, USA)^51,52^ to measure submicrometer non-refractory aerosol chemical composition. Most typically, particulate ammonium, nitrate, sulfate, chloride and organics are reported. Among the instruments are the ones equipped with a quadrupole (Q-AMS) or time-of-flight mass spectrometers in the compact version C-ToF (ref. 53) and high resolution version HR-ToF (ref. 54) which have been deployed in numerous short-term field campaigns^55,56^. For long-term observations, a monitoring type version of the AMS, the aerosol chemical speciation monitor (ACSM), has been developed^57^ with either a quadrupole (Q-ACSM) or time-of-flight^58^ (ToF-ACSM) mass spectrometer. The Q-ACSM has been used extensively and validated within the ACTRIS network stations^59^. In this work, four datasets originate from the Q-ACSM, and one each from the ToF-ACSM and HR-ToF-AMS. Below follows a joint description of all mass spectrometer types, while specificities are presented under the station headings.

In general, all AMS and ACSM types sample aerosol through a critical orifice and an aerodynamic lens system. Typically, the critical orifice has a diameter of 100 μm, restricting the sample flow to 0.08 l min^−1^. The lens system focuses the particles into a narrow beam and concentrates them before they are accelerated into a vacuum chamber and hit a vaporizer operated at 600 °C. Particles in the size range from 150 to 450 nm are transmitted by the aerodynamic lens to about 100% efficiency, while also a significant fraction of the particles between 70 and 1000 nm are transmitted^60^. The cut-offs vary slightly between instruments as they are a function of the pumping and quality of the established vacuum. Particles are flash vaporized and their gaseous fragments are ionized by electron impact ionization (70 eV), before entering the mass spectrometer for separation according to their mass-to-charge ratio. To determine the aerosol concentration, the instrumental background signal is subtracted from the ambient signal. In the case of the ACSM, a valve switching system in front of the critical orifice guides the sample flow in regular intervals through a filter which retains all particles. In case of the AMS, a chopper, installed in the vacuum chamber, is regularly moved into the beam keeping the particles from reaching the vaporizer. An additional difference in the AMS is that particles fly through a time-of-flight region extending from the chopper to the vaporizer, where the aerodynamic diameter of the particles can be measured. This type of information, however, is not included in this work as most chemical composition measurements were conducted by ACSMs and particle size distribution data from the MPSS is available. Finally, the obtained mass spectra are de-convoluted by applying a so-called fragmentation table based on laboratory experiments^61^ to report the typical inorganic and organic aerosol components. More detailed descriptions of the functioning of the various AMS and ACSM types can be found in refs 52–54,57,58.

To ensure reliable results, several well-established procedures^52,59,62^ including calibrations were routinely carried out by instrument operators. In addition, all Q-ACSMs operated within ACTRIS participated in an intercomparison exercise^59,62^ (marked with ‘*’ in Table 3) at the Aerosol Chemical Monitor Calibration Center. Generally, the latest acquisition and analysis software were used. After instrument transport, the lens system needs to be aligned such that the particle beam hits the vaporizer unit centrally and all voltages need to be tuned for optimal ion detection in the mass spectrometer. Protocols are described in refs 51,57. The instrument intercomparison described in these references took place in 2013 during many of the reported periods in this dataset. Data from this period are not reported. Also, the instrument-specific flow rates were determined regularly as this directly affects the mass concentration calculation. The mass-to-charge ratios (*m/z*) are calibrated using three or more peaks for all ToF mass spectrometers and two peaks for the Q-ACSM, typically including *m/z* 28 (N_2_^+^). This procedure was either automated with the help of the data acquisition software or carried out every couple of days.

To determine aerosol mass concentration, the ionization efficiency (*IE*) is calibrated using ammonium nitrate, and all other species’ mass concentrations are expressed in nitrate equivalent mass. Relative to the ionization efficiency of nitrate (*IE*_*NO3*_), ionization efficiencies of sulfate and ammonium (*RIE*_*SO4*_, typically between 0.6 and 1.2, *RIE*_*NH4*_, typically between 2.5 and 5 (ref. 58)) were determined. Refs 52,59 describe in detail the theory and the standard calibration procedures, the latter specifically for Q-ACSMs as suggested by the ACTRIS intercomparison facility. The *IE* and *RIEs* were determined at the beginning of the measurement and then repeated regularly. Based on the high number of field and laboratory measurements with the aerosol mass spectrometers, it is known that not all particles flash vaporize but can bounce of the heater and are hence not detected^52^. To account for this, a correction factor, called collection efficiency (*CE*), is applied. For ambient measurements, *CE* is typically ~0.5. However, factors such as particle water content or the nitrate fraction also play a role and a particle composition-dependent *CE* can be calculated^63^. The *CE* applied to each station’s dataset is provided in Table 4.

The detection limit is typically determined by sampling particle-free air through a filter. The signal oscillates around zero and the detection limit is defined as three times the standard deviation specific to a certain time resolution. Table 5 shows the one-hour detection limits for all relevant mass spectrometer types. Data below the detection limit are not reported in the datasets.

Generally, results obtained from the Q-ACSM, ToF-ACSM and HR-ToF-AMS are in good agreement with R^2^>0.9 for all species except chloride^59^. There is some uncertainty in the determination of the organic aerosol (OA) mass concentration with the Q-ACMS due to variability in the detection of the ion CO_2_^+^ at *m/z* 44. This ion typically constitutes the largest single contribution to OA (*f*_*44*_), but as discussed in a Q-ACSM intercomparison paper^62^, this *f*_*44*_ contribution can vary from 8.5 to 18.2% between instruments for the same aerosol. One factor contributing to the *f*_*44*_ variability is the generation of a CO_2_^+^ signal from the thermal decomposition of inorganic species on the instrument’s vaporizer and filament^64^.

Table 4 provides details on the chemical composition data treatment protocol which includes information on the deployed instrument model, its operation mode, and acquisition and data processing software, including the collection efficiency used to create the data record. References describing data acquisition and processing following the protocols are included. Protocols for chemical composition datasets are named ‘P_typeofmassspectrometer_nameofstation’.

### Station specific remarks

#### CES

Contrary to the other CES instruments, the Q-ACSM sampled from a 5 m high inlet with a PM_2.5_ cyclone from the top of the facility building. The inlet line was 10 m long, the total flow was 9 l min^−1^ and the sample air was dried to <40% RH. A site specific routine for the determination of the *CE* was applied based on ref. 18.

#### FIK

The ACSM was connected to the PM_10_ inlet and sampled aerosol through a PM_1_ sharp cut cyclone (BGI Inc.) at 3.5 l min^−1^. The aerosol was dried to <40% before analysis with a nafion drier (TROPOS custom-built). The applied *CE* was 0.5 for all measured species. The *RIE* for NH_4_^+^ was set to 5.15 derived from the ammonium nitrate monodisperse aerosol calibration process, while *RIE* for SO_4_^2−^ was set to 0.6, estimated by the fitting approach proposed by ref. 65. For all other species the default *RIE* values were used. The concentrations of non-sea salt particulate sulfate and ammonium were validated against results from concurrent PM_1_ filter data analyzed with ion chromatography^66^.

#### JFJ

On Jungfraujoch, a ToF-ACSM was operated^67^ which was connected to the main aerosol inlet and with a bypass flow of 3 l min^−1^ in addition to the instrument’s inlet flow of 0.14 l min^−1^. The larger sample flow resulted from replacing the standard critical orifice of 100 μm in the inlet lens system with a 130 μm orifice to keep the mass flow similar to standard temperature and pressure operation conditions. Flow rate calibrations as well as the *IE* of nitrate and *RIEs* of sulfate and ammonium were carried out monthly or bimonthly and average values were applied to the data^67^. The collection efficiency was determined to be *CE*=1 based on comparison of the submicrometer aerosol mass derived by the SMPS and equivalent black carbon data from an optical measurement^67^.

#### MHD

The HR-ToF-AMS was operated as described in ref. 68 behind the community sampling system. The concentrations of non-sea salt particulate sulfate, ammonium and nitrate were validated against results from ion chromatography^68^. Different from other locations, at MHD also the contribution of sea salt to the submicrometer aerosol mass is reported. The quantification is based on ref. 69.

#### SMR

The Q-ACSM was operated 20 m away from the CCNC and DMPS at SMR, sampling from 4 m above ground behind a PM_2.5_ cyclone to prevent dust and pollen from entering^70^. The inlet was a 1 cm outer diameter stainless steel tube operated with a 3 l min^−1^ bypass flow. The aerosol sample air was dried. The *CE* is estimated yearly from a correlation study with the particle mass derived from the number size distribution measurements, taking into account black carbon concentrations, measured separately at SMR. Organic aerosol mass is verified via comparison with a semi-continuous organic carbon/elemental carbon analyzer^71^ (Sunset Laboratory Inc.).

There are no specific remarks for MEL.

### Code availability

Table 6 provides information on the custom codes used to acquire and process the datasets.

## Data Records

The aggregated data files (Data Citation 1) are available in standardized Network Common Data Form from figshare. A link to the data is also provided on the ACTRIS secondary data portal: http://actris.nilu.no/Content/Products. The files are self-explanatory as they contain all data and metadata, i.e. information about the global attributes, dimensions and variables. A large number of data fields is included within the global attributes in each file. Many were adopted from the National Center for Atmospheric Research Research Aviation Facility file format (http://www.eol.ucar.edu/raf/software/netCDF.html) such as coordinates, time coverage, etc. Additional fields include date of file creation, contact information, acknowledgment recommendations, type of platform, instrument information, measurement uncertainty, type of environment sampled, major atmospheric influences (e.g., biomass burning) etc. Table 7 lists the set of attributes (*n*=47). The time stamp is standardized to seconds since 1 January 1970 UTC (UNIX time). Aerosol variable names are also standardized. All records are fully quality-controlled as described in section ‘Technical Validation’, invalid data have been removed and all missing data are flagged.

All data are stored in files separated by station and measurement instrument. All concentrations are corrected to standard temperature and pressure and the time stamp is the end of the averaging interval in UTC. In the NetCDF files, the version number and revision date are included.

The 11 data records from cloud condensation nuclei measurements can be found in files named ‘CCN_Instrument_Project[Database]_PlatformType_PlatformName[StationName]_Startdate_Enddate.nc’.

Table 2 describes the measurement methods, operational parameters and applied codes to create the CCN time series. The file contains the time series of CCN concentrations in cm^−3^ at each available supersaturation.

The 11 data records for particle size distribution measurements are available separately for each station named ‘NSD_Instrument_Project[Database]_PlatformType_PlatformName[StationName]_Startdate_Enddate.nc’. Table 3 provides details on the measurement instruments, operational parameters and applied codes to produce the size distribution time series. The files contain the time series of particle concentrations per size bin in the format d*N*/dlog_10_(*d*_*p*_) in cm^−3^, with *N* being the particle number concentration and *d*_*p*_ the bin diameter. The midpoint diameter of each size bin is provided in nm. From the size distribution the total number of particles was calculated and is provided in the files named ‘N_Instrument_Project[Database]_PlatformType_PlatformName[StationName]_Startdate_Enddate.nc’.

Chemical composition data from 6 stations is available in separate files named ‘Comp_Instrument_Project[Database]_PlatformType_PlatformName[StationName]_Startdate_Enddate.nc’. Table 4 lists the instrument types, operational parameters, applied codes and collection efficiencies to create the time series. The mass concentrations in μg m^−3^ for each chemical component are provided.

Most of the primary measurement data included in the aggregated NetCDF data records are archived in the primary (no harmonization of data as applied in this work) data repository EBAS (http://ebas.nilu.no) in Nasa Ames 1001 format, the required format for ACTRIS and GAW-WDCA data. The files also include comprehensive metadata and quality measures defined within ACTRIS and GAW-WDCA. All submissions of ACTRIS and GAW-WDCA near-surface data are identified in the EBAS database with a unique dataset identity number (ID-numbers). This makes potential changes and revised versions traceable; the latest revision date is included in the data files. The ID-numbers for all data available through EBAS that are also used in this aggregated data record are available upon request from EBAS. The link to the last version of data is always available online through the EBAS web interface and the ACTRIS data portal. Links to primary datasets archived in EBAS are included in Table 8 where available.

## Technical Validation

The quality of the datasets was assured and assessed based on the three steps described below.

First, all data originators confirmed that they applied SOPs and recommendations provided by ACTRIS (available after registration at http://actris.eu/) or equivalent standards or user community established best practices. An overview of the ACTRIS SOPs is included in the ACRTIS Data Management Plan: (http://www.actris.eu/Portals/46/Publications/DataCentre/ACTRIS_Data_Management_Plan.pdf). The main points of each SOPs and recommendations are described in the ‘Methods’ section. ACTRIS provides documents for the operation of aerosol inlets, and operation and data processing of aerosol size distribution and cloud condensation nuclei measurements. ACTRIS recommendations are based on or complementary to the World Meteorological Organization Global Atmosphere Watch, the Atmospheric Composition Change: a European Network, the European Monitoring and Evaluation Program and the European Supersites for Atmospheric Aerosol Research recommendations. The recommendations are harmonized across the various frameworks. Aerosol chemical composition measurements and data analysis followed procedures as described by ref. 59 in the case of the Q-ACSM, ref. 58 for the ToF-ACSM, and the best practice compendium for ACSM types as described here: https://www.psi.ch/acsm-stations/acsm-best-practice. Within the user community of the HR-ToF-AMS established procedures exist as well (http://cires1.colorado.edu/jimenez-group/wiki/index.php?title=ToF-AMS_Main). In addition, several of the particle mobility size spectrometers and aerosol mass spectrometers have participated in intercomparison exercises. Those instruments are marked with ‘*’ in Table 3 and Table 4. Deviations from the general recommendations are described in the ‘Methods’ section if applicable.

Second, in the case of not having published the data in a peer-reviewed journal, each originator checked their data manually for plausibility and time periods that do not reflect ambient values (e.g., removal of calibration periods or local contamination) as recommended by the World Data Center for Aerosols (WDCA: http://www.gaw-wdca.org/SubmitData.aspx). In case of prior publication, quality assurance has been described in the references as listed in Tables 3,4,5 and 9. Note that for all aerosol chemical data the instruments have either participated in an intercomparison exercise or the dataset has been published already. Table 9 includes also information on whether data were already publicly available on the EBAS data base as a level 1 dataset. Level 1 data means that all steps in measuring and data processing are quality assured, invalid data have been removed and the data are provided in standard temperature and pressure and in native time resolution (http://www.gaw-wdca.org/SubmitData/AdvancedDataReporting/Level1.aspx).

Third, even when operators follow SOPs and recommendations as closely as possible there is always a chance of instrumental drifts or minor malfunctioning. This can especially be the case for monitoring type observations where instruments are not constantly overseen and visits occur only several times a year. Hence, we here provide general quality check metrics. We specifically emphasize CCN data checks since there has not yet been an intercomparison or calibration exercise for this type of instrument. For the chemical composition data, we summarize the validation results from the literature since all instruments participated either in an intercomparison exercise or datasets have already been published.

### CCN and size distribution data

For all the time series of CCN data it was checked that the number concentration of CCN at higher supersaturations exceeded that of lower supersaturations. To validate that the number of activated particles is determined correctly, we compared total CCN concentrations at 1.0% supersaturation to the total particle number concentration determined from the size distribution measurements. At 1.0% supersaturation most particles activate and the concentrations from the two instruments are comparable under certain constraints.

Figure 4 compares the integrated particle size distributions with the counted activated particles for all instances in which the contribution of particles <30 nm (*N*_*30*_) is at most 10% (middle panels) and between 10 and 20% (right panels). When many small particles are present, a match on the 1:1 line is not expected, since these small particles might be below the activation diameter and are hence not counted by the CCNC. The black solid line indicates the perfect match. At most stations, many points lie within the dashed lines denoting the propagated 10% counting error of both instruments (22.4% in total) that is generally expected^38,50^. However, in many cases there is also a potential bias towards undercounting CCN as can be seen in the left panels that show the median, interquartile range, 10th and 90th percentiles as well as points beyond the 2.5th and 97.5th percentiles for the ratio of total particle number to activated particles when the contribution of *N*_*30*_ is smaller than 10% (*N*_*30<10%*_*/CCN*_*1.0*_). The box in each left panel includes information on the number of points (*pnts*), the geometric median (*GeoMean*) and the geometric standard deviation (*GSD*). The grey dashed lines indicate the 22.4% interval around the 1:1 line. The bias is only potential, because particles with a diameter slightly larger than 30 nm might not activate either, as well as larger particles that are non- or only slightly hygroscopic. This means that values of the ratios *N*_*30<10%*_*/CCN*_*1.0*_ presented here can only be partly indicative of the accuracy of the measurements. It is important to note, that all slopes are larger than one meaning that the CCNC measurements did not overestimate the particle concentration at any of these stations. The width of the interquartile range or the geometric standard deviation (because data are not necessarily normally distributed) can show how reliably and comparably the two instruments operated over time. For example, if one of the instruments drifted, the interquartile range or *GSD* would become larger. These two parameters would, however, not be impacted by a systematic stable bias in either one or both instruments.

At locations with relatively high contributions of small particles such as SMR and VAV, a larger spread can be observed together with a higher *N*_*30<10%*_*/CCN*_*1.0*_ ratio, which is expected. Note that at VAV only for a very small number of instances (86) the contribution of *N*_*30*_ is below 10% and therefore the statistical analysis of this quality check is less robust than at other stations. At FIK, where also a high *N*_*30<10%*_*/CCN*_*1.0*_ ratio is observed, it has to be taken into account that larger mineral dust particles or biomass burning aerosol are present^72^ which are less hygroscopic and may impair the comparison.

At JFJ, as mentioned in the ‘Methods’ section the CCN concentrations were underestimated by about 40% in 2012 and overestimated by roughly 30% in 2013. From a detailed comparison of the SMPS with a CPC it is known that the bias stems from the CCNC. In addition, the under- and overestimations are consistent over the range of applied supersaturations which suggests a systematic quantification error. For that reason, the dataset has been corrected as shown for 1% supersaturation in Fig. 4.

At CES the CCN number concentration is strongly underestimated. The fact that many small particles are present at this station can only partly explain the bias. Examination of the bias at various supersaturations revealed that the underestimation is a function of the applied supersaturation. At higher supersaturations the underestimation becomes larger suggesting that small particles, activating at higher supersaturation, were not sufficiently accounted for by the CCNC. Not being due to insufficient droplet growth to the detection limit of 1 μm, the bias most likely owes to particle losses in the sampling line leading to the CCNC. Since this cannot be accounted for across the various supersaturations, the dataset has not been corrected. Users must hence apply caution when using and interpreting the data. Discrepancies are as large as a factor of 2.8 in the median.

**Figure 5:**
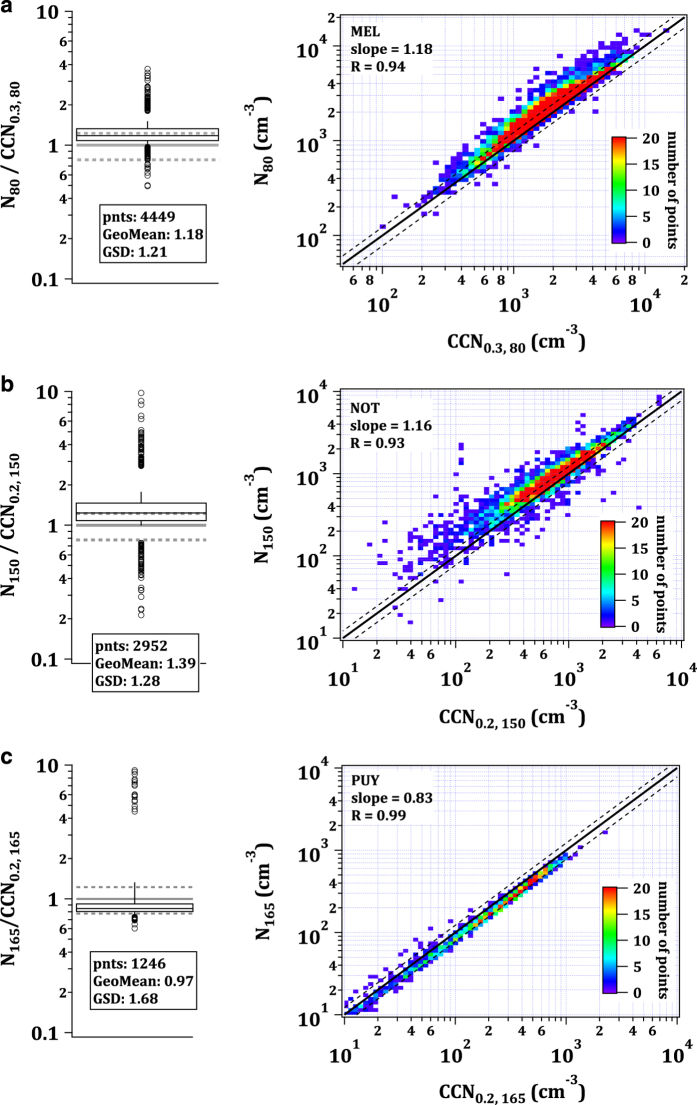
Monodisperse CCN data quality check for MEL, NOT and PUY (a), (b) and (c), respectively. Right panels: scatter plots for CCN number concentration at a certain supersaturation versus the integrated particle number from size distribution measurements greater than the indicated diameter in the subscript (in nm). The black line denotes the 1:1 line and the dashed black lines indicate the range of expected uncertainty from particle counting. Left panels: box and whiskers plots as explained in Fig. 4.

At stations with monodisperse data not including 1% supersaturation, a different quality check approach was used. Figure 5 (right panels) shows the integrated particle number size distribution above the diameter indicated in the subscript versus the integrated CCN number size distribution above the same diameter at a certain supersaturation. The selected diameter is larger than the expected activation diameter while at the same time the number of externally mixed non-hygroscopic particles is very low since these types of particles can impair the comparison. Supersaturations and cut-off diameters were chosen based on ref. 73 for PUY and ref. 29 for NOT. For Melpitz, a supersaturation of 0.3% was chosen as it represents best the supersaturation at which local clouds form. Based on long-term aerosol number size distribution measurements at the site^26,27^, the accumulation mode was found to reach down to 80 nm which was hence chosen as lower cut-off diameter. At PUY 84% of the data lie within the expected 22.4% counting uncertainty range. At Melpitz 60% lie within this range and at NOT 45%. Deviations from the 1:1 line and a spread in the data are expected due to varying ambient conditions in which the chosen cut-off diameter does not represent the actual activation diameter.

**Figure 6:**
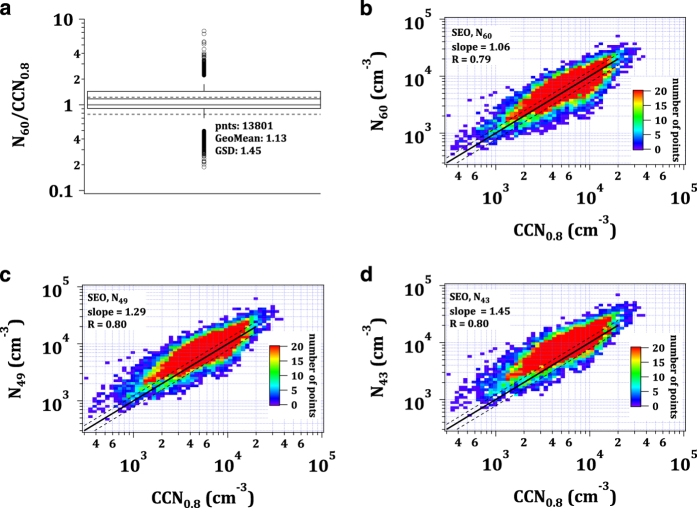
Correlation of particle number with CCN_0.8_ for SEO. Results shown in panels **b**, **c** and **d** are for N_60_, N_49_ and N_43_, respectively, using logarithmic bins with the color code indicating the number of points per bin. The slope and correlation coefficient of each fit are given in panel **a**. The black solid line is the 1:1 line, the black dashed lines indicate the 22.4% counting uncertainty range, grey solid lines indicate the 60% counting uncertainty range. The upper left panel shows the box and whiskers plot for the N_60_ to CCN_0.8_ ratio as described for Fig. 4.

A quality check for the data from SEO in the forms presented above is not possible as the maximum supersaturation was 0.8% and measurements were polydisperse. Hence we plot the *CCN*_*0.8*_ number concentration against the integrated particle number size distribution larger than 43, 49 and 60 nm, corresponding to a hygroscopicity parameter kappa of 0.3, 0.2 and 0.1, respectively (Fig. 6). A value of 0.3 has been identified as a global average for continental and urban particles^74^, while several other studies found kappa to be around 0.2, and even as low as 0.1, in urban environments with fresh emissions^75–77^. For Seoul, a low kappa value of 0.1 seems realistic as the corresponding size cut-off yields the best correlation between the datasets and the slope of the linear fit is closest to the 1:1 line within the tested range, indicating that the activation diameter is near 60 nm. The width of the interquartile range is comparable to those of other stations with polydisperse measurements, indicating that the instruments ran reliably.

### Aerosol chemical composition

For all Q-ACSM data the uncertainty in determining the total non-refractory submicrometer mass is 9%, while for the individual chemical components it is 15% for nitrate, 28% for sulfate, 36% for ammonium and 19% for organic matter^59^. Chloride concentrations were mostly near the detection limit and are not reported except for MHD, CES and FIK. For CES, correlation of chloride from the Q-ACSM to a reference instrument is, however, low with R=0.49 (ref. 17). At FIK, Q-ACSM concentrations for particulate sulfate and ammonium were compared to collocated PM_1_ filter measurements and deviated on average 19 and 15%, respectively. Particulate organics concentrations deviated on average 10% from filter based measurements^66^. The ToF-ACSM data at Jungfraujoch represents most of the time >80% of the total submicrometer particle mass derived from the SMPS data^67^. At MHD, the HR-ToF-AMS mass concentrations of non-sea salt sulfate, ammonium and nitrate were compared against external datasets and showed good agreement^68^.

## Usage Notes

The standardized NetCDF format allows for quickly loading the data into software commonly used in the atmospheric science community including the freely available software ‘R’. For more details on the format and how to access NetCDF data see http://www.unidata.ucar.edu/software/netcdf/. The files are self-explanatory as they contain all metadata and data.

The time series of the variables are designed such that they can be used without further processing. To derive the particle number concentrations from the size distributions, the area of the curve of d*N/*dlog*(D)* versus the bin diameter must be integrated taking the logarithm (log_10_) of the bin spacing into account:

$$N_{tot}=\int_{D_{min}}^{D_{max}}\frac{dN(D)}{d\log D}d\;\log\;D$$

With *N*_*tot*_=total particle number concentration, d*N*=number of particles per size bin, *D*=bin diameter. Total number concentration data is provided so users can double check their integration methods. Depending on the specific purpose, the data can also be treated with a variety of statistical analysis methods. However, users must keep in mind the uncertainties discussed in the ‘Technical Validation’ section. To determine whether atmospheric conditions were within the expected variability or exceptional for the available data record periods, we recommend users to work with complementary data such as ERA-Interim (a global atmospheric reanalysis product available from http://apps.ecmwf.int/datasets/) or similar products. For some locations, complementary data on aerosol optical properties or trace gas species can be obtained from the EBAS database (http://ebas.nilu.no/).

We encourage users to utilize the data for the following purposes (this is a non-exhaustive list):

- Detailed understanding of aerosol properties: A synthesized analysis including all variables can be produced in which aerosol characteristics can be explored and compared among different environments (e.g., remote marine versus high-altitude), throughout various seasons and large scale weather patterns (e.g., Monsoon or Arctic Haze). Also, further variables can be calculated from the datasets. For example, combining the size distribution data with the CCN number concentration, the fraction of activated particles can be derived whereby a common lower cut-off diameter (e.g., 30 nm) can be defined for maximal comparability between sites. Similarly, the particle diameter at which particles activate can be calculated following the steps as described in detail in ref. 11. Also, the hygroscopicity parameter *κ* of the bulk aerosol population can be determined, following the instructions in ref. 14.
- Improving satellite data retrievals and model representation of cloud droplet formation: Recent publications have shown that in-situ CCN data in combination with updraft information can be used to develop and improve estimates of CCN or cloud droplet number concentration from satellite observations^1,78^. The same data can be used to derive the direct sensitivity of cloud to aerosol perturbations for model and satellite evaluation^72,79^, and to understand the relative contribution of aerosol and dynamic parameters in the variability of droplet formation^80^.
- Evaluating models’ representation of aerosol properties: Recent work^4,81^ has shown how important in-situ aerosol measurements are to help constrain the uncertainty in aerosol-cloud processes related to radiative forcing. The dataset will allow the modelling community to test their results against particle number size distributions, CCN number concentration and chemical composition. Comparing global climate model output with measurements at this level of detail is necessary as the complex climate models rely on these variables for climate projections. Models that focus more on the chemical and microphysical aerosol processes as well as their transport in the atmosphere can also benefit from this dataset. In particular, the ACSM dataset can be harnessed to evaluate models’ representation of particulate organics using the volatility basis set or similar schemes. This dataset provides new data covering full annual cycles, which makes more comprehensive comparisons possibly than in previous efforts covering only very limited time periods^82–89^.

## Additional Information

**How to cite this article:** Schmale, J. *et al.* Collocated observations of cloud condensation nuclei, particle size distributions, and chemical composition. *Sci. Data* 4:170003 doi: 10.1038/sdata.2017.3 (2017).

**Publisher’s note:** Springer Nature remains neutral with regard to jurisdictional claims in published maps and institutional affiliations.

## Supplementary Material



Supplementary Material 1

## Figures and Tables

**Table 1:** Metadata record.

**Station Abbreviation**	**Geographical location/type**	**Geoposition**	**Sample**	**Protocol**
BRW	Barrow, USA, Arctic maritime	71°19’N, 156°37’W11 m	BRW_CCN	P_CCNC_BRW
			BRW_SIZE	P_size_BRW
			BRW_Ntot	P_size_BRW
CES*	Cabauw, The Netherlands, near coast, rural- background	51°58‘N, 04°56‘E−1 m	CES_CCN	P_CCNC_CES
			CES_SIZE	P_size_CES
			CES_Ntot	P_size_CES
			CES_chemistry	P_QACSM_CES
FIK*	Finokalia, Crete, Greece, coastal background, Mediterranean	35°20’N, 25°40‘E250 m	FIK_CCN	P_CCNC_FIK
			FIK_SIZE	P_size_FIK
			FIK_Ntot	P_size_FIK
			FIK_chemistry	P_QACSM_FIK
JFJ*	Jungfraujoch, Switzerland, high alpine, background	46°33’N, 07°59‘E3580 m	JFJ_CCN	P_CCNC_JFJ
			JFJ_SIZE	P_size_JFJ
			JFJ_Ntot	P_size_JFJ
			JFJ_chemistry	P_TOFACSM_JFJ
MEL*	Melpitz, Germany, continental background	51°32’N, 12°56’E,86 m	MEL_CCN	P_CCNC_MEL
			MEL_SIZE	P_size_MEL
			MEL_Ntot	P_size_MEL
			MEL_chemistry	P_QACSM_MEL
MHD*	Mace Head, Ireland, coastal background	53°20’N, 09°54‘W5 m	MHD_CCN	P_CCNC_MHD
			MHD_SIZE	P_size_MHD
			MHD_Ntot	P_size_MHD
			MHD_chemistry	P_TOFAMS_MHD
NOT	Noto Peninsula, Japan, coastal background	37°27‘N 137°22‘E0 m	NOT_CCN	P_CCNC_NOT
			NOT_SIZE	P_size_NOT
			NOT_Ntot	P_size_NOT
PUY*	Puy de Dôme, France, mountain, continental background	45°46’N, 02°57’E1465 m	PUY_CCN	P_CCNC_PUY
			PUY_SIZE	P_size_PUY
			PUY_Ntot	P_size_PUY
SEO	Seoul, South Korea, urban, monsoon-influenced	37°34′N 126°58′E38 m	SEO_CCN	P_CCNC_SEO
			SEO_SIZE	P_size_SEO
			SEO_Ntot	P_size_SEO
SMR*	Hyytiälä, Finland, rural background, boreal forest	61°51’N, 24°17‘E181 m	SMR_CCN	P_CCNC_SMR
			SMR_SIZE	P_size_SMR
			SMR_Ntot	P_size_SMR
			SMR_chemistry	P_QACSM_SMR
VAV*	Vavihill, Sweden, rural background	56°01’N, 13°09‘E172 m	VAV_CCN	P_CCNC_VAV
			VAV_SIZE	P_size_VAV
			VAV_Ntot	P_size_VAV
Stations with one asterisk form part of the ACTRIS network.				

*ACTRIS Network station.

**Table 2:** Description of cloud condensation nuclei data acquisition for each site including the instrument type, operation mode, applied supersaturations, acquisition and data processing software and references from the literature.

**Protocol Name**	**Site**	**CCNC type**	**Operation mode**	**Super-saturation (%)**	**Flow rate (l min-1)**	**Acquisition Software**	**Data processing package**	**references**
P_CCNC_BRW	Barrow (BRW)	CCN-100	polydisperse	0.20, 0.30, 0.50, 0.60, 1.00, 1.20, 1.45	0.5	standard Labview program by Droplet Measurement Technologies	Custom code	link
P_CCNC_CES	Cabauw (CES)	CCN-100	polydisperse	0.10, 0.20, 0.30, 0.50, 1.00	0.5	standard Labview program by Droplet Measurement Technologies	Custom Matlab script by ECN Environmental Assessment	8
P_CCNC_FIK	Finokalia (FIK)	CCN-100	polydisperse	0.20, 0.40, 0.60, 0.80, 1.00	0.5	standard Labview program by Droplet Measurement Technologies	Data processing Procedures within the Igor Pro 6.37 version	43
P_CCNC_JFJ	Jungfrau-joch (JFJ)	CCN-100	polydisperse	0.10, 0.15, 0.20, 0.25, 0.30, 0.35, 0.40, 0.50, 0.70, 1.00	1.0; 0.75	standard Labview program by Droplet Measurement Technologies	PSI CCNC Toolkit	11,87
P_CCNC_MEL	Melpitz (MEL)	CCN-100	monodisperse (DMA: Hauke medium TROPOS-built, CPC: TSI Model 3010)20–440 nm	0.10, 0.20, 0.30, 0.50, 0.70	0.5	TROPOS CCNC Labview program for monodisperse CCNC measurements	TROPOS CCNC software	46
P_CCNC_MHD	Mace Head (MHD)	CCN-100	polydisperse	0.10, 0.25, 0.35, 0.50, 0.75, 1.00	0.5	standard Labview program by Droplet Measurement Technologies	custom Matlab code	8
P_CCNC_NOT	Noto Peninsula (NOT)	CCN-100	monodisperse (DMA: TSI Model 3081L, CPC: TSI Model 3776)8–342 nm	0.10, 0.20, 0.50, 0.80	0.5	TSI Aerosol Instrument Manager and standard Labview program by Droplet Measurement Technologies	Scanning Mobility CCN Analysis Tool (http://nenes.eas.gatech.edu/Experiments/SMCA.html)	29
P_CCNC_PUY	Puy de Dome (PUY)	Mini-CCNC	monodisperse (TSI type DMA 44 cm, TSI CPC 3010)	0.2	0.1 with bypass flow of 0.4	LaMP custom code	LaMP custom code	
P_CCNC_SEO	Seoul (SEO)	CCN-100	polydisperse	0.20, 0.40, 0.60, 0.80	0.5	standard Labview program by Droplet Measurement Technologies	University of Seoul mysql and perl code	
P_CCNC_SMR	Hyytiälä (SMR)	CCN-100	polydisperse	0.10, 0.20, 0.30, 0.50, 1.00		standard Labview program by Droplet Measurement Technologies	University of Helsinki Matlab code	8
P_CCNC_VAV	Vavihill (VAV)	CCN-100	polydisperse	0.10, 0.15, 0.20, 0.25, 0.30, 0.35, 0.40, 0.50, 0.70, 1.00, 1.40		standard Labview program by Droplet Measurement Technologies	Lund University custom code	

**Table 3:** Description of size distribution data acquisition for each site including the instrument type, scan width and steps, acquisition and data processing software and references from the literature.

**Proto-col name**	**Site**	**mobility size measurement system**	**# of bins**	**diameter range (nm)**	**Acquisition Software**	**Data processing package**	**references**
P_size_BRW	Barrow (BRW)	TROPOS-type custom-built SMPS	33	10–810	TROPOS custom made	TROPOS custom code	50
P_size_CES*	Cabauw (CES)	SMPS TSI 3034	70	10–516	TSI standard software	Custom code	17,50
P_size_FIK	Finokalia (FIK)	TROPOS-type custom-built SMPS	71	9–849	TROPOS v4.7.2	TROPOS custom code	50
P_size_JFJ*	Jungfraujoch (JFJ)	Custom built SMPS (DMA, TSI 3071 and a CPC TSI 3775)	104	20–600	PSI Labview program	PSI SMPS Toolkit	11,22
P_size_MEL*	Melpitz (MEL)	TROPOS-type Dual SMPS custom built	46	5–800	TROPOS Labview program	TROPOS custom software	50
P_size_MHD*	Mace Head (MHD)	Custom-built SMPS (DMA TSI 3071, CPC TSI 3010, aerosol neutralizer TSI 3077)	89	25–500	custom Labview program	custom Matlab code	
P_size_NOT	Noto Peninsula (NOT)	TSI instruments (DMA: TSI Model 3081L, CPC: TSI Model 3776)8–342 nm	270	8–342	TSI Aerosol Instrument Manager	Scanning Mobility CCN Analysis Tool	29
P_size_PUY*	Puy de Dome (PUY)	Custom built DMPS (TSI type DMA 44 cm, TSI CPC 3010)	26	10–400	LaMP custom code	LaMP custom code	
P_size_SEO	Seoul (SEO)	SMPS, TSI 3936L10	106	>10–478	Standard TSI software	SMPS program by TSI, mysql and perl code	32
P_size_SMR*	Hyytiälä (SMR)	UHEL-type Custom built Dual DMPS (Hauke DMA, CPC TSI 3025A)	51	>3–1000	University of Helsinki Labview code	University of Helsinki Matlab code	88, link
P_size_VAV*	Vavihill (VAV)	ULUND-type Custom built Dual-DMPS	37	>3–900	Custom made	Custom made	35,89

*Operators at these stations participated in intercomparison workshops at the WCCAP^50^.

**Table 4:** Description of aerosol chemical data acquisition for each site including the instrument type, collection efficiency, acquisition and data processing software and references from the literature.

**Protocol name**	**site**	**aerosol mass spectrometer type**	**collection efficiency**	**Acquisition Software**	**Data processing package**	**references**
P_QACSM_CES*	Cabauw (CES)	Q-ACSM	Based on ref. 18	Aerodyne ACSM Data Acquisition and Analysis Software	Aerodyne ACSM Data Acquisition and Analysis Software	17,59
P_QACSM_FIK	Finokalia (FIK)	Q-ACSM	0.5	Aerodyne ACSM Data Acquisition and Analysis Software	Aerodyne ACSM Data Acquisition and Analysis Software	66,72
P_TOFACSM_JFJ	Jungfraujoch (JFJ)	ToF-ACSM	1	Tofwerk Acquility Software	Tofware for IGOR Pro	58,67
P_TOFAMS_MHD	Mace Head (MHD)	HR-ToF-AMS	a composition- dependent CE (Middlebrook *et al.*^63^ range: 0.45–0.97	Aerodyne AMS Data Acquisition Software	Squirrel/PIKA for IGOR Pro	
P_QACSM_MEL*	Melpitz (MEL)	Q-ACSM	Based on Middlebrook *et al.*^63^	Aerodyne ACSM Data Acquisition and Analysis Software	Aerodyne ACSM Data Acquisition and Analysis Software	59
P_QACSM_SMR*	Hyytiälä (SMR)	Q-ACSM	0.52	Aerodyne ACSM Data Acquisition and Analysis Software	Aerodyne ACSM Data Acquisition and Analysis Software	70

*These instruments participated in the 2013 intercomparison^59^.

**Table 5:** One-hour detection limits for various aerosol mass spectrometers types.

**Species**	**Q-ACSM**^57^	**ToF-ACSM**^58^	**HR-ToF-AMS**^54^
ammonium	201	23	5
organics	105	26	3
sulfate	17	2	1
nitrate	9	3	<1
chloride	8	1	2
Units are in ng m^−3^ and detection limits have been recalculated to the hour, based on equation 4 in ref. 58.			

**Table 6:** Availability of data acquisition and processing codes.

**Item**	**link**	**Accessibility**
All TROPOS custom codes (CCNC and SMPS)		Available upon request from the corresponding authors
BRW CCNC analysis code		Available upon request from the corresponding authors
All CES custom codes (CCNC, SMPS)		Available upon request from the corresponding authors
All FIK customs codes (CCNC, SMPS, ACSM)		Available upon request from the corresponding authors
All MHD custom codes/programs	http://macehead.org/	Available upon request from the corresponding authors
All PUY custom codes (CCNC, SMPS)		Available upon request from the corresponding authors
All SEO custom codes (CCNC, SMPS)		Available upon request from the corresponding authors
All SMR custom codes (CCNC, SMPS)		Available upon request from the corresponding authors
All VAV custom codes (CCNC, SMPS)		Available upon request from the corresponding authors
All PSI custom codes (CCNC Toolkit, SMPS Toolkit)		Available upon request from the corresponding authors
Scanning Mobility CCN Analysis Tool	http://nenes.eas.gatech.edu/Experiments/SMCA.html	
Aerodyne Q-ACSM acquisition and analysis software	https://sites.google.com/site/ariacsm/	Registration and login required
Aqility ToF-ACSM acquisition software	www.tofwerk.com	License purchase required
Tofware for IGOR Pro for Q- and ToF-ACSM data analysis	www.tofwerk.com	License purchase required
HR-ToF-AMS data acquisition software	https://sites.google.com/site/tofamsdaq/	From indicated webpage, registration and login required
AMS data analysis tools	http://cires1.colorado.edu/jimenez-group/ToFAMSResources/ToFSoftware/index.html	From indicated webpage, registration and login required

**Table 7:** List of attributes contained in the file metadata.

**Attribute name**	**Example content**
Additional_Data_Info	measured supersaturations spelled out as ‘0.1, 0.2, 0.3 %’
Altitude	refers to section of the atmosphere, e.g., free troposphere
Calibration_Material_Method	refers e.g., to an SOP
Cutoff_High_Diameter	40 μm
Cutoff_Low_Diameter	20 nm
Data_Contact	Email address
Data_Info	activation as CCN under various supersaturations, number concentration in cm−3
Data_Source	EBAS
Environment	Europe, alpine, high-altitude, 40% in free troposphere
Error_Absolute	NaN
Error_Bias_Correction	details described in data descriptor
Error_Characteristics	counting error, accuracy of supersaturation determination from calibration, inlet flow
Error_Relative	supersaturation±10 %, details described in data descriptor
File_ID	Syn_JFJ_CCN_2016-09-19.v1
File_N_Var	NaN
File_Var_Name	NaN
Gassp Version	1.0
GASSP Version=*	1.0
Inlet_Sample_Flow_Dry	yes
Institute	Paul Scherrer Institute
Instrument	DMT cloud condensation nuclei counter
Measurement_Mode	monitoring
Model_Serial_Number	DMT CCN-100 model
Modification_Date	2016-09-16 21:16:45.892727
Other_Inlet_Info	total aerosol 20 nm to 40 μm; Weingartner *et al.*^23^, J. Geophys. Res., 104(D21), 26,809 to 26,820
PI	Name 1, name 2,…, name n
Platform	station
Platform_Name	Jungfraujoch
Project_Name	ACTRIS
Project_URL	see data descriptor
pT_Conditions	STP
Sampling_Conditions	40 % in free troposphere
Season	all
Size_Definition	none
Software Version=	write_EBAS_ACTRIS_level1_from_level0.py
Software_Version	write_option_files_EBAS_ACTRIS.py
Species_Short_Name	total
Station_Altitude	3580
Station_ID	GAW ID JFJ
Station_Lat	49.33
Station_Lon	7.59
Time_Coordinate	NaN
Time_Coordinate=	Time
Time_Coverage_End=	31.12.2014 23:00
Time_Coverage_Start=	01.01.2012 00:00
Time_Stamp_Info	UTC, dd.mm.yyyy hh.mm.ss
Variable_Class	CCN

*GASSP refers to the Global Aerosol Synthesis and Science Project through which the data were formatted to netCDF.

**Table 8:** List of file names within Data Citation 1 and their authors.

**Sample**	**File Name**	**Authors**	**latest version on EBAS primary database**
BRW_CCN	CCN_DMT_cloud_condensation_nuclei_counter_station_Barrow_NOAA_BRW_Observatory_20.07.2007_25.06.2008.nc	Jefferson, A., Sheridan, P., Ogren, J.	
CES_CCN	CCN_DMT_cloud_condensation_nuclei_counter_station_Cesar_tower_Cabauw_ACTRIS_01.01.2012_31.12.2014.nc	Frumau, KFA, Kos, G.,Hensen, A.	
FIK_CCN	CCN_DMT_cloud_condensation_nuclei_counter_station_Finokalia_ACTRIS_01.01.2014_31.12.2015.nc	Bougiatioti, A., Mihalopoulos, N., A., Nenes, A.	
JFJ_CCN	CCN_DMT_cloud_condensation_nuclei_counter_station_Jungfraujoch_ACTRIS_01.01.2012_31.12.2014.nc	Hammer, E., Schmale, J., Motos, G., Gysel, M.	link
MEL_CCN	CCN_DMT_cloud_condensation_nuclei_counter_station_Melpitz_ACTRIS_01.01.2012_31.12.2014.nc	Henning, S., Stratmann, F.	link
MHD_CCN	CCN_DMT_cloud_condensation_nuclei_counter_station_Mace_Head_ACTRIS_01.01.2011_31.12.2012.nc	Ovadnevaite, J., O’Dowd, C.	
NOT_CCN	CCN_DMT_cloud_condensation_nuclei_counter_station_NOTO_Groundbased_Research_Observatory_nan_01.05.2014_28.02.2015.nc	Iwamoto, Y., Kinouchi, K., Matsuki, A.	
PUY_CCN	CCN_mini_cloud_condensation_nuclei_counter_station_Puy_de_Dome_ACTRIS_01.01.2014_01.01.2015.nc	Picard, D., Sellegri, K.	
SEO_CCN	CCN_DMT_cloud_condensation_nuclei_counter_station_Seoul,_South_Korea_nan_01.01.2006_31.12.2010.nc	Yum, S.S., Park, M.	
SMR_CCN	CCN_DMT_cloud_condensation_nuclei_counter_station_Smear_II_station,_Hyytiälä_ACTRIS_01.01.2012_31.12.2014.nc	Paramonov, M., Aalto, P., Keskinen, H., Petäjä, T., Kulmala, M.	link
VAV_CCN	CCN_DMT_cloud_condensation_nuclei_counter_station_Vavihill_ACTRIS_20.12.2012_11.11.2014.nc	Krisstenson, A., Wittborn, C., Svenningsson, Frank, G., B., Swietlicki, E.	link
BRW_SIZE	NSD_custom_built_scanning_mobility_particle_sizer_station_Barrow_NOAA_BRW_Observatory_20.07.2007_25.06.2008.nc	Birmili, W., Jefferson, A., Ogren, J.	
BRW_Ntot	N_custom_built_scanning_mobility_particle_sizer_station_Barrow_NOAA_BRW_Observatory_20.07.2007_25.06.2008.nc	Birmili, W., Jefferson, A., Ogren, J.	
CES_SIZE	NSD_custom_built_scanning_mobility_particle_sizer_station_Cesar_tower_Cabauw_ACTRIS_01.01.2012_31.12.2014.nc	Henzing, J.S.	link
CES_Ntot	N_custom_built_scanning_mobility_particle_sizer_station_Cesar_tower_Cabauw_ACTRIS_01.01.2012_31.12.2014.nc	Henzing, J.S.	
FIK_SIZE	NSD_custom_built_scanning_mobility_particle_sizer_-TROPOS_type_station_Finokalia_ACTRIS_01.01.2014_31.12.2015.nc	Kalivitis, N., Mihalopoulos, N.	link
FIK_Ntot	N_custom_built_scanning_mobility_particle_sizer_-TROPOS_type_station_Finokalia_ACTRIS_01.01.2014_31.12.2015.nc	Kalivitis, N., Mihalopoulos, N.	
JFJ_SIZE	NSD_custom_built_scanning_mobility_particle_sizer_station_Jungfraujoch_ACTRIS_01.01.2012_31.12.2014.nc	Herrmann, E., Bukowiecki, N., Collaud Coen, M., Gysel, M.	link
JFJ_Ntot	N_custom_built_scanning_mobility_particle_sizer_station_Jungfraujoch_ACTRIS_01.01.2012_31.12.2014.nc	Herrmann, E., Bukowiecki, N., Collaud Coen, M., Gysel, M.	
MEL_SIZE	NSD_custom_built_tandem_scanning_mobility_particle_sizer_station_Melpitz_ACTRIS_01.01.2012_31.12.2014.nc	Sonntag, A., Wiedensohler, A.	link
MEL_Ntot	N_custom_built_tandem_scanning_mobility_particle_sizer_station_Melpitz_ACTRIS_01.01.2012_31.12.2014.nc	Sonntag, A., Wiedensohler, A.	
MHD_SIZE	NSD_custom_built_scanning_mobility_particle_sizer_station_Mace_Head_ACTRIS_01.01.2011_31.12.2012.nc	Ovadnevaite, J., O’Dowd, C.	link
MHD_Ntot	N_custom_built_scanning_mobility_particle_sizer_station_Mace_Head_ACTRIS_01.01.2011_31.12.2012.nc	Ovadnevaite, J., O’Dowd, C.	
NOT_SIZE	NSD_scanning_mobility_particle_sizer_station_NOTO_Groundbased_Research_Observatory_nan_01.05.2014_28.02.2015.nc	Iwamoto, Y., Kinouchi, K., Matsuki, A.	
NOT_Ntot	N_scanning_mobility_particle_sizer_station_NOTO_Groundbased_Research_Observatory_nan_01.05.2014_28.02.2015.nc	Iwamoto, Y., Kinouchi, K., Matsuki, A.	
PUY_SIZE	NSD_custom_built_differential_mobility_particle_sizer_station_Puy_de_Dome_ACTRIS_01.01.2014_01.01.2015.nc	Picard, D., Sellegri, K., Nicolas, J.	link
PUY_Ntot	N_custom_built_differential_mobility_particle_sizer_station_Puy_de_Dome_ACTRIS_01.01.2014_01.01.2015.nc	Picard, D., Sellegri, K., Nicolas, J.	
SEO_SIZE	NSD__scanning_mobility_particle_sizer_station_Seoul,_South_Korea_nan_01.01.2006_31.12.2010.nc	Yum, S.S., Park, M.	
SEO_Ntot	N__scanning_mobility_particle_sizer_station_Seoul,_South_Korea_nan_01.01.2006_31.12.2010.nc	Yum, S.S., Park, M.	
SMR_SIZE	NSD_custom_built_differential_mobility_particle_sizer_station_Smear_II_station,_Hyytiälä_ACTRIS_01.01.2012_31.12.2014.nc	Aalto, P., Keskinen, H., L., Petäjä, T., Kulmala, M.	link
SMR_Ntot	N_custom_built_differential_mobility_particle_sizer_station_Smear_II_station,_Hyytiälä_ACTRIS_01.01.2012_31.12.2014.nc	Aalto, P., Keskinen, H., L., Petäjä, T., Kulmala, M.	
VAV_SIZE	NSD_custom_built_twin_differential_mobility_particle_sizer_station_Vavihill_ACTRIS_20.12.2012_11.11.2014.nc	Krisstenson, A., Wittborn, C., Svenningsson, Frank, G., B., Swietlicki, E.	link
VAV_Ntot	N_custom_built_twin_differential_mobility_particle_sizer_station_Vavihill_ACTRIS_20.12.2012_11.11.2014.nc	Krisstenson, A., Wittborn, C., Svenningsson, Frank, G., B., Swietlicki, E.	
CES_chemistry	Comp_quadrupol_aerosol_chemical_speciation_monitor_station_Cesar_tower_Cabauw_ACTRIS_01.01.2012_31.12.2014.nc	Schlag, P., Frumau, A., Holzinger, R., Kiendler-Scharr, A.	link
FIK_chemistry	Comp_quadrupol_aerosol_chemical_speciation_monitor_station_Finokalia_ACTRIS_01.01.2014_31.12.2015.nc	Stavroulas, I., Bougiatioti, A., Mihalopoulos, N.	link
JFJ_chemistry	Comp_time-of-fligh_aerosol_chemical_speciation_monitor_station_Jungfraujoch_ACTRIS_01.01.2012_31.12.2014.nc	Fröhlich, R.	link
MEL_chemistry	Comp_quadrupole_aerosol_chemical_speciation_monitor_station_Melpitz_ACTRIS_01.01.2012_31.12.2014.nc	Poulain, L.	link
MHD_chemistry	Comp_high_resolution_time-of-fligh_aerosol_mass_spectrometer_station_Mace_Head_ACTRIS_01.01.2011_31.12.2012.nc	Ovadnevaite, J., O’Dowd, C.	link
SMR_chemistry	Comp_quadrupole_aerosol_chemical_speciation_monitor_station_Smear_II_station,_Hyytiälä_ACTRIS_01.01.2012_31.12.2014.nc	Heikkinen, L., Äijälä, M., Keskinen, H., Aalto, P., Petäjä, T., Kulmala, M., Ehn, M.	link
Links are provided for the corresponding primary datasets on the EBAS data base where available. ‘Primary dataset’ in this context means that the data were submitted to EBAS prior to creating this combined dataset. The primary data might have a different time resolution and a different temporal coverage.			

**Table 9:** Overview of datasets that have already been published, that were obtained from instruments that passed an intercomparison exercise at the World Calibration Center for Aerosol Physics (WCCAP) or that were obtained from instruments that were compared to an instrument that passed an intercomparison exercise.

**Station**	**CCNC**			**Mobility particle spectrometer**			**Aerosol mass spectrometer**		
	Inter-comparison*	**publication**	**available in EBAS**†	**Intercomparison**†	**publication**	**available on EBAS**‡	**Inter-comparison**	**publication**	**available in EBAS**‡
BRW	n.a.			n.a.			no instrument		
CES	n.a.			participated, 2009 (ref. 50)		yes	passed, Dec. 2013^59^		yes
FIK	n.a.		yes	June 2009 on-site auditingSeptember 2013 passed lab intercomparisonJanuary 2016 passed lab intercomparison		yes	with collocated PM_1_ filter data		yes
JFJ	n.a.			participated, 2009 (ref. 50) passed, Jul. 2011, May 2014		yes	does not apply (prototype instrument)		yes
MEL	n.a.		yes	(ref. 50), 2011 good on-site intercomparison, May/Jun. 2013 passed lab intercomparison, Jul. 2013 installation of new TSMPS after passed intercomparison, Aug. 2013 removal of old TDMPS, agreement between old and new within required boundaries, 2015 passed on-site intercomparison		yes	passed, Dec. 2013 ^59^, and Mar. 2016		yes
MHD	n.a.			participated, 2009 (ref. 50) recommendations for improvement received Oct. 2012		yes	does not apply (different instrument type)	^68^,§	yes
NOT	n.a.			n.a.			data not yet processed		
PUY	n.a.			passed, Oct. 2013		yes	no instrument		
SEO	n.a.			n.a.			no instrument		
SMR	n.a.		yes	participated, 2009 (ref. 50) passed, May 2016		yes	passed, Dec. 2013 (ref. 59), and Mar. 2016		yes
VAV	n.a.			passed, Oct. 2013		yes	no instrument		
Note that intercomparisons for CCNCs were not introduced before 2016 (denoted as n.a.=not available).									

*the first CCNC intercomparison will take place in fall 2016 at the European Center for Aerosol Calibration.

^†^reports are available from the European Center for Aerosol Calibration (http://www.actris-ecac.eu/contact.php).

^‡^as of 24 August 2016 as a primary dataset on http://actris.nilu.no/, archived in EBAS.

^§^ref. 68 describes the data quality and instrument reliability for the site, but does not discuss the dataset included here as it covers an earlier time period.
