# Corrigendum: Collocated observations of cloud condensation nuclei, particle size distributions, and chemical composition

**DOI:** 10.1038/sdata.2018.94

**Published:** 2018-05-08

**Authors:** Julia Schmale, Silvia Henning, Bas Henzing, Helmi Keskinen, Karine Sellegri, Jurgita Ovadnevaite, Aikaterini Bougiatioti, Nikos Kalivitis, Iasonas Stavroulas, Anne Jefferson, Minsu Park, Patrick Schlag, Adam Kristensson, Yoko Iwamoto, Kirsty Pringle, Carly Reddington, Pasi Aalto, Mikko Äijälä, Urs Baltensperger, Jakub Bialek, Wolfram Birmili, Nicolas Bukowiecki, Mikael Ehn, Ann Mari Fjæraa, Markus Fiebig, Göran Frank, Roman Fröhlich, Arnoud Frumau, Masaki Furuya, Emanuel Hammer, Liine Heikkinen, Erik Herrmann, Rupert Holzinger, Hiroyuki Hyono, Maria Kanakidou, Astrid Kiendler-Scharr, Kento Kinouchi, Gerard Kos, Markku Kulmala, Nikolaos Mihalopoulos, Ghislain Motos, Athanasios Nenes, Colin O’Dowd, Mikhail Paramonov, Tuukka Petäjä, David Picard, Laurent Poulain, André Stephan Henry Prévôt, Jay Slowik, Andre Sonntag, Erik Swietlicki, Birgitta Svenningsson, Hiroshi Tsurumaru, Alfred Wiedensohler, Cerina Wittbom, John A. Ogren, Atsushi Matsuki, Seong Soo Yum, Cathrine Lund Myhre, Ken Carslaw, Frank Stratmann, Martin Gysel

*Scientific Data* 4:170003 doi: 10.1038/sdata.2017.3 (2018); Published 14 March 2017; Updated 8 May 2018

The affiliation of Cathrine Lund Myhre was incorrectly stated in the original publication. It is: 19 NILU -Norwegian Institute for Air Research, Instituttveien 18, Kjeller 2007, Norway.

